# A Sonotrombólise Promove Melhora dos Índices de Motilidade e Perfusão do Ventrículo Esquerdo após o Infarto Agudo do Miocárdio

**DOI:** 10.36660/abc.20200651

**Published:** 2022-04-07

**Authors:** Bruno G. Tavares, Miguel Osman Aguiar, Jeane Tsutsui, Mucio Oliveira, Alexandre de Matos Soeiro, José Nicolau, Henrique Ribeiro, Hsu PoChiang, João Sbano, Carlos Eduardo Rochitte, Bernardo Lopes, José Ramirez, Roberto Kalil, Wilson Mathias

**Affiliations:** 1 Hospital das Clínicas Faculdade de Medicina Universidade de São Paulo São Paulo SP Brasil Instituto do Coração do Hospital das Clínicas da Faculdade de Medicina da Universidade de São Paulo , São Paulo , SP – Brasil

**Keywords:** Infarto do Miocárdio, Sonotrombólise, Microbolhas, Meios de Contraste, Função Ventricular Esquerda, Embolia Pulmonar

## Abstract

**Fundamento:**

Demonstrou-se recentemente que a aplicação de ultrassom de alta energia com microbolhas, técnica conhecida como sonotrombólise, causa a dissolução de trombos intravasculares e aumenta a taxa de recanalização angiográfica no infarto agudo do miocárdio com supradesnivelamento do segmento ST (IAM-CSST).

**Objetivo:**

Avaliar o efeito da sonotrombólise nos índices de motilidade e perfusão miocárdicas em pacientes com IAM-CSST, utilizando a ecocardiografia com perfusão miocárdica em tempo real (EPMTR).

**Método:**

Uma centena de pacientes com IAM-CSST foram randomizados em dois grupos: Terapia (50 pacientes tratados com sonotrombólise e angioplastia coronária primária) e Controle (50 pacientes tratados com angioplastia coronária primária). Os pacientes realizaram EPMTR para analisar a fração de ejeção do ventrículo esquerdo (FEVE), o índice de escore de motilidade segmentar (IEMS) e o número de segmentos com defeito de perfusão miocárdica, 72 horas após o IAM-CSST e com 6 meses de acompanhamento. Foi considerado significativo p < 0,05.

**Resultados:**

Pacientes tratados com sonotrombólise apresentaram FEVE mais alta que o grupo Controle em 72 horas (50 ± 10% *vs.* 44 ± 10%; p = 0,006), e essa melhora foi mantida em seis meses (53 ± 10% *vs.* 48 ± 12%; p = 0,008). O IEMS foi similar nos grupos Terapia e Controle em 72 horas (1,62 ± 0,39 *vs.* 1,75 ± 0,40; p = 0,09), mas tornou-se menor no grupo Terapia em 6 meses (1,46 ± 0,36 *vs.* 1,64 ± 0,44; p = 0,02). O número de segmentos com defeito de perfusão não foi diferente entre os grupos em 72 horas (5,92 ± 3,47 *vs.* 6,94 ± 3,39; p = 0,15), mas ficou menor no grupo Terapia em 6 meses (4,64 ± 3,31 *vs.* 6,57 ± 4,29; p = 0,01).

**Conclusão:**

A sonotrombólise em pacientes com IAM-CSST resulta na melhora dos índices de motilidade e perfusão ventricular ao longo do tempo.

## Introdução

No Brasil, as doenças cardiovasculares são responsáveis por aproximadamente 28% de todos os óbitos anuais, metade deles por síndromes coronarianas agudas. ^1^ As terapias disponíveis atualmente para recanalização no infarto agudo do miocárdio incluem fibrinólise farmacológica e intervenção coronária percutânea, as quais têm melhorado o prognóstico de pacientes. Infelizmente, no Brasil, tais técnicas estão disponíveis apenas para cerca de 40% da população. Ainda assim, quando o paciente é submetido a uma destas terapias de eleição, a ocorrência de *no-reflow* (morte celular extensa na área infartada) está presente em aproximadamente 60% dos pacientes tratados. ^2^

A restauração da patência da artéria coronária, o mais rapidamente possível, é determinante e tem consequências importantes nos resultados de melhora da qualidade e quantidade de vida e da redução de internações hospitalares e dos custos ao sistema de saúde. ^3 - 6^

A sonotrombólise é uma terapia inovadora, que consiste na infusão endovenosa contínua de microbolhas, associada à aplicação intermitentemente de ultrassom de alta energia, o que resulta em ruptura das microbolhas e na lise de trombo intravascular. ^7 - 9^ Uma potencial aplicação da sonotrombólise, demonstrada em estudos experimentais, é destinada à recanalização de artéria coronária no contexto de infarto agudo do miocárdio (IAM). ^9^ Apesar da ampla base de estudos em animais, poucas análises tentaram demonstrar a sua eficácia em seres humanos. Uma primeira tentativa ocorreu pelo uso isolado do ultrassom na recanalização de artérias epicárdicas em pacientes com IAM, no estudo PLUS, sem sucesso. ^10^ Uma experiência inicial em número restrito de pacientes, também por feita por Slikkerveer e colaboradores no IAM, demonstrou exequibilidade e ausência de complicações. ^11^ Nosso grupo demonstrou, de forma pioneira e em 30 pacientes com infarto agudo do miocárdio e supradesnivelamento do segmento ST (IAM-CSST), que a sonotrombólise é uma terapia segura e resulta em aumento da recanalização angiográfica e melhora da microcirculação coronariana. ^12^ Mais recentemente, realizamos o estudo *Microvascular Recovery with Ultrasound in Acute Myocardial Infarction* (MRUSMI), ^13^ desenhado para investigar os efeitos clínicos da aplicação de ultrassom diagnóstico, com alto índice mecânico associado a microbolhas, em 100 pacientes com IAM-CSST randomizados em grupo controle e que receberam terapia com sonotrombólise. Nesse estudo, publicado recentemente, demonstrou-se que os pacientes tratados com o procedimento antes e imediatamente após a angioplastia coronária primária apresentaram maior taxa de recanalização coronária pré-angioplastia e menor tamanho de infarto, o que foi constatado através de ressonância magnética.

A Ecocardiografia com Perfusão Miocárdica em Tempo Real (EPMTR) é uma técnica que permite a análise simultânea da motilidade segmentar e da perfusão do ventrículo esquerdo, e tem sido utilizada para diagnóstico e avaliação de prognóstico de pacientes com doença arterial coronariana. ^14 - 17^ Como os efeitos da sonotrombólise nos índices de motilidade e perfusão a longo prazo ainda não foram estudados, propomos avaliar seu efeito nos índices de motilidade e número de segmentos com defeito de perfusão miocárdica passados 72 horas e seis meses do tratamento de pacientes IAM-CSST, utilizando a EPMTR.

## Método

### Protocolo de estudo

Os 100 pacientes desse estudo fazem parte do ensaio Recuperação Microvascular com Ultrassom no Infarto Agudo do Miocárdio ( *Microvascular Recovery with Ultrasound in Acute Myocardial Infarction* – MRUSMI; Clinical Trials.gov # NCT02410330), que foi desenhado para investigar se a aplicação de impulsos de alto índice mecânico (IM) de um transdutor de ultrassom diagnóstico durante a infusão de microbolhas comercialmente disponíveis em pacientes com IAM-CSST aumentaria precocemente as taxas de patência epicárdica e o fluxo microvascular. ^13^ Trata-se de um ensaio clínico randomizado e prospectivo. Os critérios de exclusão do estudo foram: infarto agudo do miocárdio prévio, cardiomiopatia conhecida, doença valvar significativa, uso de terapia fibrinolítica antes da chegada ao departamento de emergência, alergia ao contraste ecocardiográfico Definity ^®^ e dor precordial maior que 12 horas na chegada.

**Figura 1:**
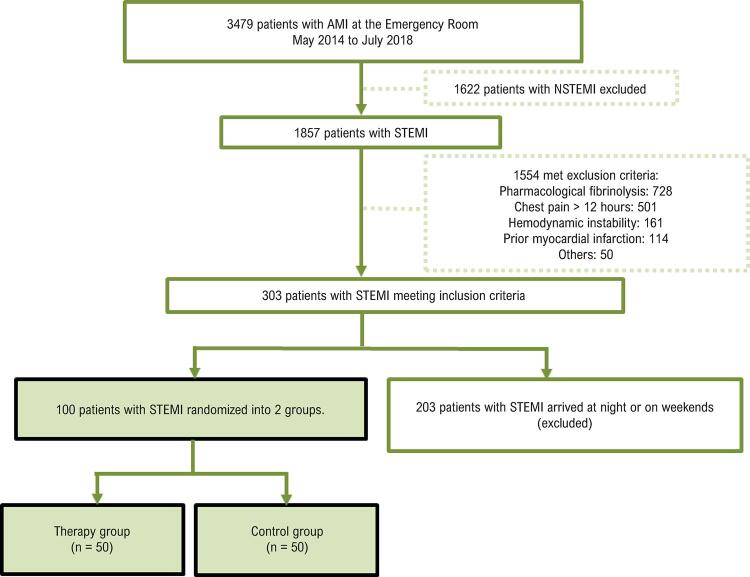
– Fluxograma do estudo MRUSMI (Microvascular Recovery with Ultrasound in Acute Myocardial Infarction).

Entre maio de 2014 e julho de 2018, 3.479 pacientes com IAM-CSST chegaram ao Departamento de Emergência da nossa instituição. Destes, 303 indivíduos apresentavam critérios de inclusão para o protocolo de estudo e 100 pacientes chegaram quando o ultrassom diagnóstico de emergência podia ser aplicado antes e após a intervenção coronária percutânea (período das 7h às 19h, de segunda a sexta-feira), conforme demonstrado na Figura 1 . Os 100 pacientes com IAM-CSST foram randomizados de forma aleatória através de *site* específico (www.random.org, plano de randomização #4544). O procedimento foi simples, mantido sob os cuidados exclusivos da enfermeira-coordenadora do estudo e desconhecido de todos os participantes até o momento de aceite do paciente em participar do mesmo.

Todos os pacientes receberam tratamento medicamentoso de acordo com o protocolo da instituição e com as diretrizes de tratamento de IAM-CSST. ^6^ Os pacientes do grupo Terapia (n = 50) receberam ultrassom diagnóstico com múltiplos impulsos de alto IM guiados por imagem (1,8 MHz; índice mecânico 1,1-1,3; duração de pulso de 3 useg), aplicados nas janelas apicais 4, 2 e 3 câmaras. O *frame rate* foi de 25 Hz. O ultrassom foi realizado com infusão de microbolhas comercialmente disponíveis (5% Definity ^®^ ) a 1,5 ml/min. Os impulsos de alto IM foram aplicados durante breves intervalos, repetidamente, após imagens de baixo IM detectarem microbolhas na microvasculatura miocárdica. Os intervalos entre os impulsos de alto IM variaram de 5 a 15 segundos, dependendo do tempo necessário para o repreenchimento miocárdico pelo contraste. Os pacientes do grupo controle (n = 50) realizaram ecocardiograma com imagens diagnósticas, usando um transdutor de ultrassom diagnóstico de 1,8 MHz com imagens de baixo IM (0,18) e *frame rate* de 25 Hz limitados, não mais que 3, e impulsos diagnósticos de alto IM para avaliar a motilidade regional de parede e a perfusão microvascular antes e após a intervenção coronária percutânea. O ultrassom foi realizado com infusão de microbolhas comercialmente disponíveis (5% Definity ^®^ ) a 1,5 ml/min.

**Figura 2:**
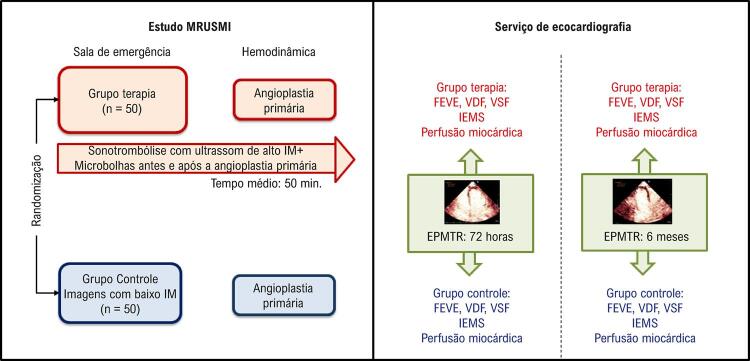
– Protocolo de estudo. Os pacientes avaliados participaram do ensaio MRUSMI (Microvascular Recovery with Ultrasound in Acute Myocardial Infarction), randomizados para receber tratamento com sonotrombólise associada a angioplastia coronária primária (grupo terapia) ou tratamento convencional com angioplastia coronária primária (grupo controle). Os pacientes de ambos os grupos realizaram Ecocardiografia com Perfusão Miocárdica em Tempo Real (EPMTR) 72 horas e 6 meses após a randomização, para avaliação de volumes ventriculares, função sistólica e perfusão miocárdicas. IM: índice mecânico; FEVE: fração de ejeção do ventrículo esquerdo; VDF: volume diastólico final; VSF: volume sistólico final; IEMS: índice de escore de motilidade segmentar.

**Figura 3:**
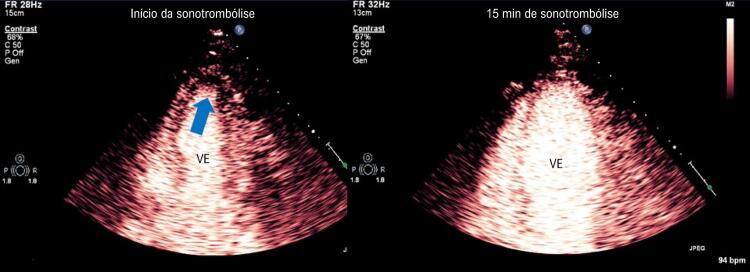
– Imagem de perfusão miocárdica em tempo real demonstrando defeito de perfusão em região apical do ventrículo esquerdo em paciente com infarto agudo do miocárdio e supradesnivelamento do segmento ST antes do início da sonotrombólise (imagem à esquerda, seta). Após 15 minutos da sonotrombólise, houve desaparecimento do defeito de perfusão miocárdica. O paciente apresentou recanalização angiográfica com a sonotrombólise.

Para fins de avaliação do índice de escore de motilidade segmentar (IEMS) e número de segmentos com defeito de perfusão miocárdica ao longo do tempo, todos os pacientes realizaram EPMTR 72 horas após a randomização e em seis meses de acompanhamento ( Figura 2 ). A Figura 3 ilustra exemplo de imagem do ventrículo esquerdo em 2 câmaras com defeito de perfusão apical antes da aplicação da sonotrombólise. Com 15 minutos de procedimento, houve desaparecimento do defeito de perfusão miocárdica.

Esse estudo foi aprovado pelo Comitê de Ética em Pesquisa da Faculdade de Medicina da Universidade de São Paulo (CAPPesq), sob o protocolo n ^o^ 0578/11. Todos os procedimentos envolvidos nesse estudo estão de acordo com a Declaração de Helsinque de 1975, atualizada no ano de 2013. O consentimento informado foi obtido de todos os participantes incluídos no estudo.

### Ecocardiografia com Perfusão Miocárdica em Tempo Real (EPMTR)

O estudo ecocardiográfico foi realizado com equipamento IE 33 (Philips Medical Systems, Bothell, WA, USA), equipados com transdutores transtorácicos de banda larga com 2-5 MHz e *software* de perfusão miocárdica. O foco foi fixado ao nível da valva mitral em todos os estudos. O ventrículo esquerdo foi avaliado em três planos ecocardiográficos padrões: apicais quatro, duas e três câmaras, definindo-se 17 segmentos, de acordo com as recomendações do *Cardiac Imaging Committe of the Concil on Clinical Cardiology of the American Heart Association* . ^18^ Todos os ecocardiogramas foram analisados em *software* específico Q-Station 3.2.2. (Philips Medical Systems, Bothell, WA, USA) após o adequado armazenamento digital. Para a análise da perfusão miocárdica, foram adquiridas imagens ecocardiográficas através de *software* específico de imagem com perfusão miocárdica em tempo real. As imagens foram ajustadas antes da injeção do contraste para minimizar os artefatos, em decorrência da mobilidade cardíaca. Uma sequência de pulsos ultrassônicos, com utilização de IM elevado e maior que 1,0 ( *Flash* ), foram disparados manualmente no pico de intensidade do contraste para destruir microbolhas dentro do miocárdio. Na sequência, foram analisadas as imagens com baixo índice mecânico (0,1) pelo período de, ao menos, 15 ciclos cardíacos consecutivos para permitir o posterior repreenchimento miocárdico. O paciente avaliado apresentou recanalização angiográfica. Para medir a intensidade de sinal pela EPMTR, sequências representativas de imagens precedendo e seguindo a imagem de *flash* foram digitalmente capturadas, armazenadas em disco óptico e posteriormente analisadas. Imagens diagnósticas de baixo IM com contraste ultrassonográfico foram utilizadas para avaliar a perfusão microvascular, motilidade regional de parede e fração de ejeção do ventrículo esquerdo (FEVE) 72 horas após a randomização e em seis meses de acompanhamento ( Figura 2 ).

### Avaliação da motilidade segmentar e perfusão miocárdicas

As imagens contrastadas foram usadas para calcular medidas da FEVE, volume diastólico final e volume sistólico final pelo método biplanar de Simpson, de acordo com as diretrizes da Sociedade Americana de Ecocardiografia. ^19^ O índice de escore de motilidade segmentar (IEMS) foi avaliado por meio de análise do espessamento da parede de cada segmento miocárdico em todas as três janelas apicais realçadas por contraste, e foi calculado através da somatória do valor dado a cada segmento (1 = contratilidade normal, 2 = hipocinesia, 3 = acinesia e 4 = discinesia), dividida pelo número total de segmentos analisados. A análise da perfusão miocárdica foi realizada usando um sistema de escore, sendo considerado o índice 1 para repreenchimento do contraste no miocárdio em 4 segundos da aplicação do impulso de alto IM, o escore 2 (ligeira redução) quando o repreenchimento completo na área de risco demorou mais que 4 segundos após o impulso de alto IM, e o escore 3, definido como praticamente sem repreenchimento de contraste miocárdio durante 10 segundos após o impulso de alto IM. Um escore de 3 foi considerado obstrução microvascular. ^16^ Para análises comparativas entre os grupos terapia e controle, foram avaliados o número de segmentos miocárdicos com escore 2 ou 3 no período de 72 horas após o tratamento e em seis meses de acompanhamento.

Todas as avaliações de FEVE, motilidade de parede e perfusão microvascular foram feitas por um revisor ecocardiografista experiente e independente (WMJ), de forma cega ao tratamento atribuído no momento das mensurações. O profissional não tinha conhecimento da sequência de randomização, que foi aberta somente após o término das análises da FEVE, motilidade de parede e perfusão microvascular. Em estudo publicado anteriormente, houve validação da variabilidade intraobservador para medidas do volume diastólico final (correlação intraclasse de 0,949; p < 0,001), volume sistólico final (correlação intraclasse de 0,987; p < 0,001) e FEVE (correlação intraclasse de 0,817; p < 0,001). ^13^

### Análise estatística

O cálculo amostral foi realizado com base em dados do estudo-piloto ^12^ e amostra de 100 pacientes, considerando 20% de possíveis perdas, visando alcançar significância estatística com p < 0,05 e poder de 80% através de premissas comparativas entre os grupos terapia e controle na resolução do segmento ST de 80% *vs.* 50%, aumento da patência angiográfica precoce, em pelo menos 50% *vs.* 20% e redução de 30% na área de infarto pela ressonância magnética.

As variáveis categóricas foram apresentadas em tabelas, descrevendo suas frequências absolutas (n) e relativas (%). O teste de qui-quadrado ou teste exato de Fisher foram usados para avaliar sua associação. As variáveis contínuas foram apresentadas em tabelas descrevendo suas médias e desvio-padrão. O teste de Kolmogorov-Smirnov avaliou se a distribuição era normal. Nos dois grupos de pacientes randomizados, mudanças no IEMS, número de segmentos com defeito de perfusão e FEVE entre os momentos 72 horas e 6 meses foram comparados através de uso de teste t não pareado. As comparações entre os momentos 6 meses e 72 horas, nos grupos terapia e controle, foram realizadas pelo teste t Student pareado. Todas as análises foram realizadas com o auxílio de SPSS 17.0 para Windows. Foi considerado estatisticamente significativo p < 0,05.

## Resultados

A média etária dos pacientes randomizados foi de 59 anos e não houve diferença em relação ao sexo nos grupos estudados. Também não houve diferença na prevalência de diabetes, hipertensão arterial, dislipidemia e tabagismo ( Tabela 1 ). A distribuição do território arterial do IAM-CSST foi semelhante nos grupos controle e terapia ( Tabela 2 ).

**Tabela 1:** – Características clínicas dos pacientes dos grupos Controle e Terapia

Variáveis	Total	Grupos		p

		Controle	Terapia	
Idade (anos)	59,06 + 10,39	59,04 + 11,01	59,08 + 9,85	0,985 ^(1)^
Altura (cm)	167,70 + 8,47	169,04 + 8,30	166,36 + 8,51	0,114 ^(1)^
Peso (Kg)	75,49 + 16,23	76,61 + 16,32	74,40 + 16,24	0,501 ^(1)^
ASC (m ^2^ )	1,84 + 0,22	1,87 + 0,22	1,82 + 0,22	0,313 ^(1)^
Sexo masculino	72 (72,0%)	40 (80,0%)	32 (64,0%)	0,075 ^(2)^
ICP prévia	8 (8,0%)	3 (6,0%)	5 (10,0%)	0,715 ^(3)^
Tabagismo	44 (44,0%)	20 (40,0%)	24 (48,0%)	0,20 ^(2)^
Dislipidemia	35 (35,0%)	15 (30,0%)	20 (40,0%)	0,295 ^(2)^
Diabetes	32 (32,0%)	11 (22,0%)	21 (42,0%)	0,032 ^(2)^
Hipertensão	56 (56,0%)	28 (56,0%)	28 (56,0%)	1,000 ^(2)^
Medicação em uso				
Ácido acetilsalicílico	98 (98,0%)	50 (100,0%)	48 (96,0%)	0,495 ^(3)^
Estatina	33 (33,0%)	14 (28,0%)	19 (38,0%)	0,288 ^(2)^
Nitrato	52 (52,0%)	25 (50,0%)	27 (54,0%)	0,689 ^(2)^
Betabloqueador	19 (19,0%)	5 (10,0%)	14 (28,0%)	0,022 ^(2)^
Bloqueador de canal de cálcio	9 (9,0%)	4 (8,0%)	5 (10,0%)	1,000 ^(3)^
IECA	20 (20,0%)	9 (18,0%)	11 (22,0%)	0,617 ^(2)^

*Variáveis expressas como média ± desvio-padrão ou número (%). ^(1)^ Teste t Student não pareado; ^(2)^ teste de qui-quadrado; ^(3)^ teste exato de Fisher. ASC: área de superfície corporal; ICP: intervenção coronária percutânea; IECA: inibidor de enzima de conversão de angiotensina.*

**Tabela 2:** – Distribuição de território arterial do infarto agudo do miocárdio com supradesnivelamento do segmento ST

Variáveis	Grupo controle	Grupo terapia	Valor de p
ADA	26 (52%)	26 (52%)	0,83 ^(1)^
ACD	14 (28%)	17 (34%)	
ACX	10 (20%)	7 (14%)	

*Variáveis expressas como número (%). ^(1)^ Teste de qui-quadrado. ADA: artéria coronária descendente anterior; ACD: artéria coronária direita; ACX: artéria coronária circunflexa.*

A Tabela 3 demonstra os valores de volumes ventriculares e FEVE da população total e dos grupos controle e terapia, nos momentos 72 horas e após 6 meses da randomização. O grupo que recebeu sonotrombólise (grupo terapia) apresentou menores volumes diastólico e sistólico final e maior FEVE que o grupo controle, 72 horas após o IAM-CSST. Todos os pacientes realizaram EPMTR no acompanhamento, sendo que essa diferença foi mantida em 6 meses de acompanhamento.

**Tabela 3:** – Volumes e fração de ejeção obtidos pela ecocardiografia com perfusão miocárdica em tempo real 72 horas e 6 meses após a randomização

Variáveis	Total	Grupos		p (entre grupos controle e terapia)

		Controle	Terapia	
72 horas				
VDF (mL)	108 ± 35	114 ± 40	102 ± 29	0,096 ^(1)^
VSF (mL)	59 ± 30	66 ± 34	53±23	0,022 ^(1)^
FEVE (%)	47 ± 11	44 ± 11	50±10	0,006 ^(1)^
IEMS	1,68 ± 0,39	1,75 ± 0,40	1,62 ± 0,39	0,09 ^(1)^
#Segmentos com defeito de perfusão	6,42 ± 3,49	5,92 ± 3,47	6,94 ± 3,39	0,15 ^(1)^
6 meses				
VDF (mL)	122 ± 47	136 ± 52*	109 ± 36	0,003 ^(1)^
VSF (mL)	66 ± 39	76 ± 45*	55 ± 29	0,006 ^(1)^
FEVE (%)	50 ± 12	47 ± 12*	53 ± 10*	0,008 ^(1)^
IEMS	1,52 ± 0,37	1,64 ± 0,44*	1,46 ± 0,36*	0,02 ^(1)^
#Segmentos com defeito de perfusão	5,86 ± 3,84	6,57 ± 4,29	4,64 ± 3,31*	0,01 ^(1)^

Variáveis expressas como média ± desvio-padrão. ^(1)^ Teste t Student não pareado entre grupos controle e terapia. *p < 0,05 pelo teste t Student pareado (comparação dos parâmetros entre 6 meses e 72 horas). VDF: volume diastólico final; VSF: volume sistólico final; FEVE: fração de ejeção do ventrículo esquerdo; IEMS: índice de escore de motilidade segmentar.

**Figura 4:**
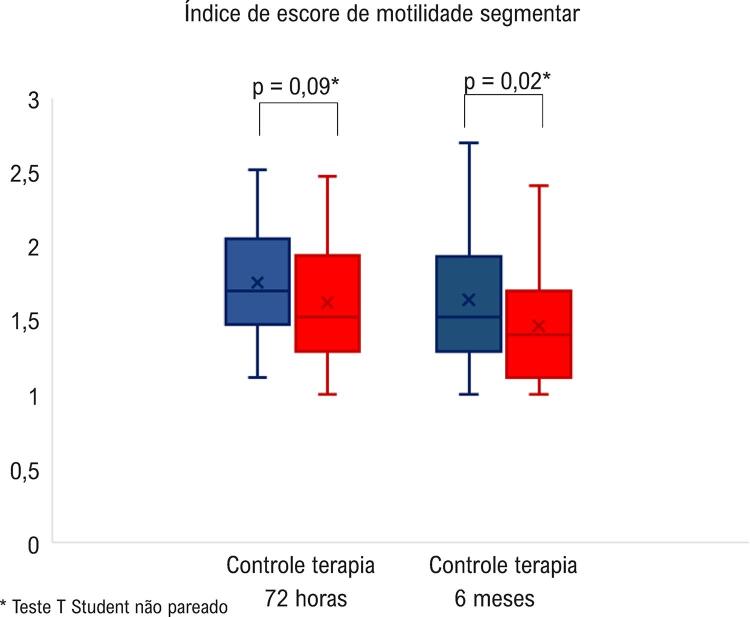
– Índice de escore de motilidade segmentar nos grupos controle e terapia, avaliados pela ecocardiografia com perfusão miocárdica em tempo real 72 horas e 6 meses após a randomização.

**Figura 5:**
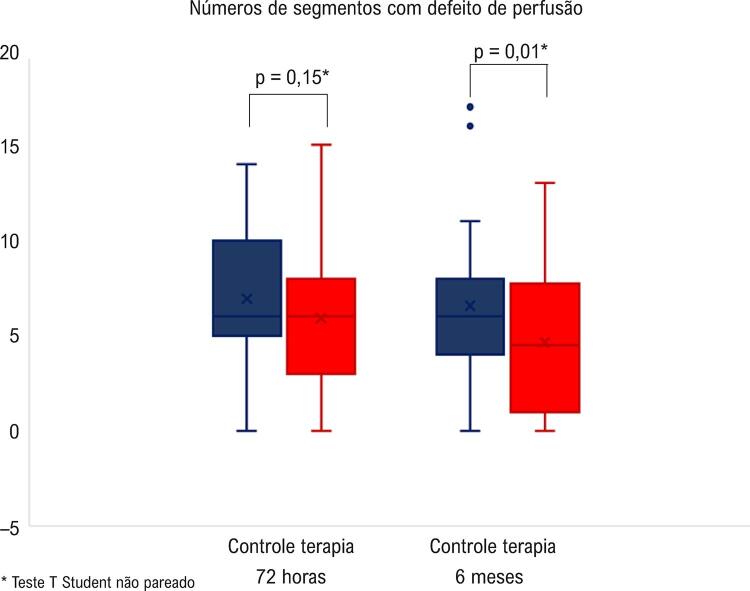
– Número de segmentos com defeito de perfusão nos grupos Controle e Terapia, avaliados pela ecocardiografia com perfusão miocárdica em tempo real 72 horas e 6 meses após a randomização.

Não houve diferença significativa entre os grupos terapia e controle em relação ao IEMS no momento 72 horas (1,62 ± 0,39 *vs.* 1,75 ± 0,40; p = 0,09). No entanto, após 6 meses de acompanhamento, o grupo Terapia evoluiu com menor IEMS que o grupo controle (1,46 ± 0,36 *vs.* 1,64 ± 0,44; p = 0,02), como demonstrado na Figura 4 . O menor valor de IEMS demonstra melhora da função ventricular esquerda. Em relação à perfusão miocárdica obtida pela EPMTR, não foi observada diferença entre o número de segmentos com defeito de perfusão entre os grupos terapia e controle 72 horas após o IAM-CSST (5,92 ± 3,47 *vs.* 6,94 ± 3,39; p = 0,15). Entretanto, após 6 meses de acompanhamento, o grupo terapia apresentou menor número de segmentos com defeito de perfusão que o grupo controle (4,64 ± 3,31 *vs.* 6,57 ± 4,29; p = 0,01), como demonstrado na Figura 5 . No período médio de 17 meses, 8 pacientes (16%) morreram no grupo controle e 8 pacientes (16%) no grupo terapia.

* Teste T Student não pareado

## Discussão

Este é o primeiro estudo em humanos que avaliou o efeito da sonotrombólise na função e perfusão ventricular esquerda em acompanhamento de 6 meses após o IAM-CSST. Utilizando a EPMTR, demonstramos que os pacientes com IAM-CSST e tratados com essa nova terapia apresentaram melhora ao longo do tempo do IEMS e do número de segmentos com defeito de perfusão miocárdica. Os resultados do estudo MRUSMI demonstraram que os tempos porta-balão não foram diferentes entre os grupos controle e terapia (78 ± 32 min *vs.* 77 ± 26 min, respectivamente; p = 0,42). A recanalização do vaso culpado no primeiro angiograma, antes da angioplastia primária, foi observada em 24/50 (48%) pacientes do grupo Terapia, em comparação com 10/50 (20%) do grupo Controle (p < 0,001). O grupo Terapia apresentou menor tamanho de infarto pela ressonância magnética, realizada 72 horas após o IAM-CSST, que o grupo Controle (29 ± 22 gramas *vs.* 40 ± 20 gramas; p = 0.026). ^13^

Tais efeitos benéficos foram evidentes a nível microvascular, a melhora do fluxo capilar foi observada imediatamente após a intervenção coronária percutânea. Até então, não havia sido avaliado o comportamento do IEMS e o número de segmentos com defeito de perfusão miocárdica ao longo do tempo. Nossos resultados confirmam que a recanalização precoce e a melhora da microcirculação coronária obtidas com a sonotrombólise possuem benefícios adicionais aos pacientes com IAM-CSST, quando comparadas aos pacientes que receberam tratamento convencional com angioplastia primária.

O ultrassom transtorácico com alta energia tem sido estudado como um tratamento adjuvante aos fibrinolíticos na abordagem de trombos arteriais, bem como um método isolado no tratamento de trombos vasculares. ^20 , 21^ Um mecanismo proposto de como o ultrassom dissolve o trombo é induzir a cavitação, ^22 , 23^ que é a geração ultrassônica dos corpos de gases que expandem, retraem e leva a forças de cisalhamento, que perturbam o meio e possuem o potencial de romper trombos. Estudos que se utilizam de sistemas baseados em cateteres, capazes de liberar ultrassom na artéria coronária, provaram-se capazes de dissolver trombos sem o uso de um agente fibrinolítico. Esse tipo de sistema, de baixa frequência ultrassônica (45 KHz) e alta energia liberadas através da ponta de um cateter de 1,6 milímetros, demonstrou recanalizar com sucesso a artéria descendente anterior de doentes que sofreram um infarto agudo do miocárdio de parede anterior. ^24^ A fim de superar as limitações do ultrassom nas síndromes coronarianas agudas, estudos experimentais têm demonstrado que a associação da administração de microbolhas sob o efeito do ultrassom pode acelerar a dissolução de trombos. Microbolhas de gás são pequenas microesferas, que apresentam propriedades acústicas específicas e tornam-se muito úteis como agentes de contraste ultrassonográfico para o diagnóstico por imagem. Ao agirem como núcleos de cavitação, as microbolhas reduzem o limiar de pico de pressão negativa necessário para induzir a mesma. Dessa forma, a destruição de microbolhas mediada por ultrassom pode acelerar ainda mais a dissolução de trombos. Em modelos animais de trombose da artéria ilíaca, o ultrassom transcutâneo de baixa frequência, associado a microbolhas injetadas via intravenosa, produziram taxas de recanalização de mais de 90%, sem a necessidade de um agente trombolítico. ^25^ Em um estudo pré-clínico em 45 porcos, demonstrou-se que, durante uma infusão intravenosa contínua de microbolhas que contém perfluorocarbonos, a energia ultrassônica emitida por um transdutor de ultrassom diagnóstico é capaz de restaurar o fluxo da microcirculação e melhorar as taxas de recanalização de artérias coronárias. ^26^ Um ensaio clínico randomizado (PLUS – *Perfusion by Thrombolytic and Ultrasound* ) que procurou avaliar o valor adicional do ultrassom terapêutico, sem microbolhas e apenas em pacientes com infarto agudo do miocárdio, foi interrompido. ^10^ Recomendaram a interrupção do estudo em julho de 2003 por causa da baixa probabilidade de diferenças significativas no grau de fluxo coronário pelo escore de TIMI ( *Trombolysis in Myocardial Infarction* ) ou pela resolução do segmento ST com o tratamento pelo ultrassom. Hoje, sabemos que a causa do insucesso deste estudo foi possivelmente a falta da associação do ultrassom intermitente com as microbolhas. Sem a presença dessas, não há cavitação inercial tissular e liberação de óxido nítrico suficientes, a fim de promover sonotrombólise e redução do *no-reflow* de forma eficaz. ^10^ Mathias et al., ^12^ em 2016, publicaram estudo-piloto da avaliação de 30 pacientes, em que foi demonstrada a segurança e a exequibilidade da aplicação de ultrassom com alto IM e infusão contínua de microbolhas para recanalização precoce e melhora da microcirculação coronária em pacientes com IAM-CSST. ^12^ Tais achados foram confirmados no estudo MRUSMI, ampliando a população para 100 pacientes. ^13^ Os impulsos de alto IM, utilizados para melhorar a recanalização epicárdica e microvascular no atual estudo, são parte de um recurso-padrão em sistema ultrassonográfico e normalmente usado para avaliar a perfusão miocárdica e a motilidade regional de parede. ^14 - 17^ Os impulsos de alto IM causam cavitação nas microbolhas (aumento e colapso) durante o período de insonação, que finalmente as rompem. ^9^ Tal crescimento e colapso causam tensão de cisalhamento em regiões próximas às microbolhas, que, no caso de um trombo, resulta em dissolução.

Os motivos pelos quais a sonotrombólise pode resultar em melhora do IEMS e de perfusão miocárdica em 6 meses pode estar associada a vários fatores, ainda não conhecidos totalmente. O principal fator parece ser a recanalização precoce das artérias coronárias, antes da realização da intervenção percutânea, observada no estudo MRUSMI (48% no grupo Terapia *vs.* 20% no grupo Controle). Uma menor área de infarto, também pela ressonância foi observada em 72 horas no grupo Terapia. Outro possível efeito poderia estar relacionado à indução de liberação de óxido nítrico. ^27^ Novos estudos multicêntricos são necessários para esclarecer os mecanismos fisiopatológicos e comprovar os benefícios da sonotrombólise em pacientes com síndromes coronarianas agudas. Vale ressaltar o potencial dessa nova opção terapêutica para o tratamento de condições trombóticas agudas. ^28 , 29^

### Limitações do estudo

Como o presente estudo foi uma subanálise do ensaio MRUSMI, os demais dados relacionados aos resultados angiográficos, e os dados eletrocardiográficos e relacionados aos biomarcadores cardíacos foram previamente relatados. Nossos resultados foram limitados aos achados da EPMTR, com foco na análise do IEMS e no número de segmentos com defeito de perfusão miocárdica. Entretanto, enfatizamos o ineditismo dos achados e a importância desses efeitos em 6 meses de acompanhamento dos pacientes com IAM-CSST tratados com sonotrombólise. Trata-se de um estudo unicêntrico, realizado com um pequeno número de pacientes e que deve ser ampliado para avaliações multicêntricas, a fim de comprovar os achados dessa iniciativa pioneira. Outro ponto que poderia ser levantado como limitação do estudo é a subjetividade da análise da motilidade segmentar e da perfusão miocárdica. Entretanto, ressaltamos a ampla aplicação de tais índices na rotina da prática ecocardiográfica, e que os pesquisadores envolvidos no estudo apresentam grande experiência na técnica de EPMTR. ^30 - 32^

## Conclusão

A sonotrombólise é uma nova abordagem terapêutica no tratamento de pacientes com IAM-CSST, que resulta em melhora do índice de motilidade de parede do ventrículo esquerdo e redução do defeito de perfusão ao longo do tempo.
