# Supplementary material for: A Sonotrombólise Promove Melhora dos Índices de Motilidade e Perfusão do Ventrículo Esquerdo após o Infarto Agudo do Miocárdio
Source: Arq Bras Cardiol. 2022 Apr 7;118(4):756–65. [Article in Portuguese] doi: 10.36660/abc.20200651 (PMC9007009; doi:10.36660/abc.20200651)
Supplement: Translation

# Translation: Sonothrombolysis Promotes Improvement in Left Ventricular Wall Motion and Perfusion Scores after Acute Myocardial Infarction

**Authors:** Bruno G. Tavares, Miguel Osman Aguiar, Jeane Tsutsui, Mucio Oliveira, Alexandre de Matos Soeiro, José Nicolau, Henrique Ribeiro, Hsu PoChiang, João Sbano, Carlos Eduardo Rochitte, Bernardo Lopes, José Ramirez, Roberto Kalil, Wilson Mathias

**Affiliations:** 1 Brazil Instituto do Coração do Hospital das Clínicas da Faculdade de Medicina da Universidade de São Paulo, São Paulo, SP – Brazil

**Keywords:** Myocardial Infarction, Sonothrombolysis, Microbubbles, Contrast Media, Ventricular Function Left, Pulmonary Embolism

## Abstract

**Background:**

It has recently been demonstrated that the application of high-energy ultrasound and microbubbles, in a technique known as sonothrombolysis, dissolves intravascular thrombi and increases the angiographic recanalization rate in patients with ST-segment–elevation myocardial infarction (STEMI).

**Objective:**

To evaluate the effects of sonothrombolysis on left ventricular wall motion and myocardial perfusion in patients with STEMI, using real-time myocardial perfusion echocardiography (RTMPE).

**Methods:**

One hundred patients with STEMI were randomized into the following 2 groups: therapy (50 patients treated with sonothrombolysis and primary coronary angioplasty) and control (50 patients treated with primary coronary angioplasty). The patients underwent RTMPE for analysis of left ventricular ejection fraction (LVEF), wall motion score index (WMSI), and number of segments with myocardial perfusion defects 72 hours after STEMI and at 6 months of follow-up. P < 0.05 was considered statistically significant.

**Results:**

Patients treated with sonothrombolysis had higher LVEF than the control group at 72 hours (50% ± 10% versus 44% ± 10%; p = 0.006), and this difference was maintained at 6 months of follow-up (53% ± 10% versus 48% ± 12%; p = 0.008). The WMSI was similar in the therapy and control groups at 72 hours (1.62 ± 0.39 versus 1.75 ± 0.40; p = 0.09), but it was lower in the therapy group at 6 months (1.46 ± 0.36 versus 1.64 ± 0.44; p = 0.02). The number of segments with perfusion defects on RTMPE was similar in therapy and control group at 72 hours (5.92 ± 3.47 versus 6.94 ± 3.39; p = 0.15), but it was lower in the therapy group at 6 months (4.64 ± 3.31 versus 6.57 ± 4.29; p = 0.01).

**Conclusion:**

Sonothrombolysis in patients with STEMI resulted in improved wall motion and ventricular perfusion scores over time.

## Introduction

In Brazil, cardiovascular diseases are responsible for approximately 28% of all deaths annually, half of which are due to acute coronary syndromes. ^1^ The therapies that are currently available for recanalization in acute myocardial infarction (AMI) include pharmacological fibrinolysis and percutaneous coronary intervention, which show improved prognosis of patients with AMI. Unfortunately, in Brazil, these techniques are only available to approximately 40% of the population. Nevertheless, if the patient is treated by one of these choice therapies, the phenomenon of no-reflow (extensive cellular death in the infarcted area) occurs in approximately 60% of patients treated. ^2^

Restoring the patency of the coronary artery as quickly as possible is decisive, and it has important consequences in the results of improved quality of life and longevity, reduced hospitalization, and reduced costs to the health system. ^3 - 6^

Sonothrombolysis is an innovative therapy, which consists of continuous intravenous infusion of microbubbles associated with intermittent application of high-energy ultrasound, resulting in rupture of the microbubbles and lysis of the intravascular thrombus. ^7 - 9^ A potential application of sonothrombolysis that has been demonstrated in experimental studies is recanalization of the coronary artery in the context of AMI. ^9^ In spite of a wide array of studies in animals, few studies have attempted to demonstrate the efficacy of sonothrombolysis in human beings. An initial attempt took place with the use of ultrasound alone in the recanalization of epicardial arteries in patients with AMI, which was unsuccessfully tested in the Perfusion by Thrombolytic and Ultrasound (PLUS) trial. ^10^ In an initial experience in a restricted number of patients, Slikkerveer et al. also demonstrated its feasibility and absence of complications in patients with AMI. ^11^ In a groundbreaking manner, our group has demonstrated, in 30 patients with ST-segment–elevation myocardial infarction (STEMI), that sonothrombolysis is a safe therapy and that it results in increased angiographic recanalization and improved coronary microcirculation. ^12^ More recently, we carried out the Microvascular Recovery with Ultrasound in Acute Myocardial Infarction (MRUSMI) trial, designed to investigate the clinical effects of the application of diagnostic ultrasound with a high mechanical index (MI), associated with microbubbles in 100 patients with STEMI, who were randomized into a control group and a therapy group of patients who received sonothrombolysis. This recently published study ^13^ demonstrated that the patients who were treated with sonothrombolysis before and immediately after primary coronary angioplasty showed greater pre-angioplasty coronary recanalization rate and reduced infarct size, as demonstrated by magnetic resonance.

Real-time myocardial perfusion echocardiography (RTMPE) is a technique that allows for simultaneous analysis of left ventricular wall motion and perfusion, which has been used for diagnosis and prognostic evaluation of patients with coronary artery disease. ^14 - 17^ As the effects of sonothrombolysis on long-term wall motion and perfusion scores have not yet been studied, we proposed an evaluation of the effect of sonothrombolysis on scores that measure wall motion and number of segments with myocardial perfusion defects 72 hours and 6 months after treatment of patients with STEMI, using RTMPE.

## Methods

### Study Protocol

The 100 patients in this study are part of the MRUSMI trial (ClinicalTrials.gov # NCT02410330), which was designed to investigate whether the application of high-MI impulses, from a diagnostic ultrasound transducer, during the infusion of commercially available microbubbles, in patients with STEMI, would increase early epicardial patency rates and microvascular flow. ^13^ This is a randomized, prospective clinical trial. Exclusion criteria for the study were prior AMI, known cardiomyopathy, significant valve disease, use of fibrinolytic therapy before arrival at the emergency department, allergy to Definity® echocardiographic contrast, and chest pain lasting more than 12 hours upon arrival.

From May 2014 to July 2018, 3479 patients with STEMI arrived at the emergency department of our institution. Of these, 303 met the inclusion criteria for the study protocol, and 100 arrived at a time when it was possible to apply the emergency diagnostic ultrasound before and after percutaneous coronary intervention (7:00 am to 7:00 pm, Monday through Friday), as shown in Figure 1 . The 100 patients with STEMI were randomized, using a randomization website (www.random.org, randomization plan # 4544). Simple randomization was used. It was kept under the exclusive care of the nurse who coordinated the study, and it was unknown to all participants until the moment the patients accepted to participate in the study.

**Figure 1:** – Flowchart of the Microvascular Recovery with Ultrasound in Acute Myocardial Infarction (MRUSMI) trial. AMI: acute myocardial infarction; NSTEMI: non–ST-segment–elevation myocardial infarction; STEMI: ST-segment–elevation myocardial infarction.

All patients received medical treatment in accordance with the institutional protocol, which follows STEMI treatment guidelines. ^6^ Patients in the therapy group (n = 50) received diagnostic ultrasound with multiple image-guided, high-MI impulses (1.8 MHz; MI 1.1 to 1.3; 3 µsec pulse duration) applied in the apical 4-, 2-, and 3-chamber views. The frame rate was 25 Hz. The ultrasound was performed with an infusion of commercially available microbubbles (5% Definity®) at 1.5 ml/min. The high-MI pulses were applied repeatedly during brief intervals, after low-MI images had detected microbubbles in the myocardial microvasculature. The intervals between high-MI impulses ranged from 5 to 15 seconds, depending on the time required for myocardial contrast replenishment. The patients in the control group (n = 50) underwent echocardiography with diagnostic images using a 1.8-MHz diagnostic ultrasound transducer with low-MI (0.18) images, frame rate of 25 Hz, and limited (no more than 3), high-MI diagnostic impulses to evaluate regional wall motion and microvascular perfusion before and after percutaneous coronary intervention. Ultrasound was performed with an infusion of commercially available microbubbles (5% Definity®) at 1.5 ml/min.

In order to evaluate wall motion score index (WMSI) and the number of segments with myocardial perfusion defects over time, all patients underwent RTMPE 72 hours after randomization and at 6 months of follow-up ( Figure 2 ). Figure 3 illustrates an example of an image of the left ventricle in 2- chamber view with apical perfusion defect before the application of sonothrombolysis. After 15 minutes of sonothrombolysis, the myocardial perfusion defect disappeared.

**Figure 2:** – Study protocol. The evaluated patients participated in the Microvascular Recovery with Ultrasound in Acute Myocardial Infarction (MRUSMI) trial, randomized to receive treatment with sonothrombolysis associated with primary coronary angioplasty (therapy group) or conventional treatment with primary coronary angioplasty (control group). Patients in both groups underwent real-time myocardial perfusion echocardiography (RTMPE) 72 hours and 6 months after randomization, for evaluation of ventricular volumes, systolic function, and myocardial perfusion. EDV: end-diastolic volume; ESV: end-systolic volume; LVEF: left ventricular ejection fraction; MI: mechanical index; WMSI: wall motion score index.

**Figure 3:** – Real-time myocardial perfusion imaging demonstrating perfusion defect in the apical region of the left ventricle of a patient with ST-segment–elevation myocardial infarction before initiating sonothrombolysis (left image, arrow). After 15 minutes of sonothrombolysis, the myocardial perfusion defects disappeared. The patient showed angiographic recanalization with sonothrombolysis. LV: left ventricle.

This study was approved by the Research Ethics Committee of the Faculty of Medicine of the University of São Paulo (CAPPesq, acronym in Portuguese), under protocol number 0578/11. All procedures involved in this study are in accordance with the Declaration of Helsinki of 1975, updated in 2013. Informed consent was obtained from all participants included in the study.

### Real-time myocardial perfusion echocardiography (RTMPE)

Echocardiography study was conducted with IE 33 equipment (Philips Medical Systems, Bothell, WA, USA), equipped with wide-band, 2-to-5–MHz, transthoracic transducers and myocardial perfusion software. In all studies, focus was set at the level of the mitral valve. The left ventricle was evaluated in 3 standard echocardiographic planes, namely, apical 4-, 2-, and 3-chamber views, defining 17 segments, as recommended by the Cardiac Imaging Committee of the Council on Clinical Cardiology of the American Heart Association. ^18^ All echocardiograms were analyzed on Q-Station 3.2.2 specific software (Philips Medical Systems, Bothell, WA, USA) after appropriate digital storage. For analysis of myocardial perfusion, echocardiography images were acquired with specific imaging software with real-time myocardial perfusion. The images were adjusted before injection of contrast to minimize the artifacts due to cardiac mobility. A sequence of ultrasound pulses, with the use of elevated MI, greater than 1.0 (flash), were manually fired at the peak of contrast intensity to destroy microbubbles within the myocardium. Then, images with low MI (0.1) were analyzed for at least 15 consecutive cardiac cycles to allow for subsequent myocardial replenishment. The patient showed angiographic recanalization. To measure signal strength via RTMPE, representative sequences of images preceding and following the flash image were digitally captured, stored on an optical disk, and analyzed at a later moment. Diagnostic low-MI images with ultrasound contrast were used to assess microvascular perfusion, regional wall motion, and left ventricular ejection fraction (LVEF), 72 hours after randomization and at 6 months of follow-up ( Figure 2 ).

### Evaluation of myocardial wall motion and perfusion

The contrasted images were used to calculate measurements of LVEF, end-diastolic volume, and end-systolic volume, using Simpson’s biplane method, in accordance with the guidelines of the American Society of Echocardiography. ^19^ The WMSI was evaluated by analyzing the wall thickening of each myocardial segment in all 3 apical windows, highlighted by contrast. This score was calculated by summing the values attributed to each segment (1 = normal kinesia, 2 = hypokinesia, 3 = akinesia, and 4 = dyskinesia), divided by the total number of segments analyzed. Analysis of myocardial perfusion was carried out using a scoring system, where myocardial contrast replenishment within 4 seconds of application of high-MI impulse was assigned a value of 1; when complete replenishment in the risk area took more than 4 seconds after the application of high-MI impulse, a value of 2 (mild reduction) was assigned; and, when there was practically no myocardial contrast replenishment for 10 seconds after the high-MI impulse, a value of 3 was assigned. A score of 3 was considered microvascular obstruction. ^16^ For comparative analysis between the therapy and control groups, the number of myocardial segments with scores of 2 or 3 was evaluated at 72 hours after treatment and at 6 months of follow-up.

All evaluations of LVEF, wall motion, and microvascular perfusion were carried out by an experienced, independent echocardiographic reviewer (WMJ), who was blinded to the treatment given at the moment of the measurements. The reviewer knew nothing about the randomization sequence, which was opened only after the conclusion of analyses of LVEF, wall motion, and microvascular perfusion. In a previously published study, intraobserver variability was validated for measurements of end-diastolic volume (intraclass correlation of 0.949; p < 0.001), end-systolic volume (intraclass correlation of 0.987; p < 0.001), and LVEF (intraclass correlation of 0.817; p < 0.001). ^13^

### Statistical Analysis

The sample calculation was based on data from the pilot study. ^12^ A sample size of 100 patients was calculated, including 20% possible losses, to achieve statistical significance of p < 0.05 and power of 80%, using comparative assumptions between the therapy and control groups, ST-segment resolution of 80% versus 50%, an increase in early angiographic patency of at least 50% versus 20%, and a 30% reduction in the infarcted area on magnetic resonance imaging.

Categorical variables were shown in tables describing their absolute (n) and relative (%) frequency, and chi-square test or Fisher’s exact test was used to evaluate their association. Continuous variables were shown in tables describing their means and standard deviation. The Kolmogorov-Smirnov test evaluated whether distribution was normal. In both groups of randomized patients, changes in WMSI, number of segments with perfusion defects, and LVEF between 72 hours and 6 months were compared using the unpaired t test. Comparisons between 6 months and 72 hours, in the therapy and control groups, were made by paired Student t test. All analyses were carried out with the assistance of SPSS 17.0 for Windows. P < 0.05 was considered statistically significant.

## Results

Average age of randomized patients was 59 years, and there were no differences in relation to sex between the study groups. There were also no differences regarding prevalence of diabetes, high blood pressure, dyslipidemia, and tobacco use ( Table 1 ). The distribution of the STEMI arterial territory was similar in the control and therapy groups ( Table 2 ).

**Table 1:** – Clinical characteristics of patients in the control and therapy groups

Variables	Total	Groups		p

		Control	Therapy	
Age (years)	59.06 + 10.39	59.04 + 11.01	59.08 + 9.85	0.985 ^(1)^
Height (cm)	167.70 + 8.47	169.04 + 8.30	166.36 + 8.51	0.114 ^(1)^
Weight (kg)	75.49 + 16.23	76.61 + 16.32	74.40 + 16.24	0.501 ^(1)^
BSA (m2)	1.84 + 0.22	1.87 + 0.22	1.82 + 0.22	0.313 ^(1)^
Male sex	72 (72.0%)	40 (80.0%)	32 (64.0%)	0.075 ^(2)^
Prior PCI	8 (8.0%)	3 (6.0%)	5 (10.0%)	0.715 ^(3)^
Tobacco use	44 (44.0%)	20 (40.0%)	24 (48.0%)	0.20 ^(2)^
Dyslipidemia	35 (35.0%)	15 (30.0%)	20 (40.0%)	0.295 ^(2)^
Diabetes	32 (32.0%)	11 (22.0%)	21 (42.0%)	0.032 ^(2)^
Hypertension	56 (56.0%)	28 (56.0%)	28 (56.0%)	1.000 ^(2)^
Medication use				
Aspirin	98 (98.0%)	50 (100.0%)	48 (96.0%)	0.495 ^(3)^
Statins	33 (33.0%)	14 (28.0%)	19 (38.0%)	0.288 ^(2)^
Nitrate	52 (52.0%)	25 (50.0%)	27 (54.0%)	0.689 ^(2)^
Betablocker	19 (19.0%)	5 (10.0%)	14 (28.0%)	0.022 ^(2)^
Calcium channel blocker	9 (9.0%)	4 (8.0%)	5 (10.0%)	1.000 ^(3)^
ACEI	20 (20.0%)	9 (18.0%)	11 (22.0%)	0.617 ^(2)^

*Variables expressed as mean ± standard deviation or number (%). ^(1)^ Unpaired Student’s t test; ^(2)^ Chi-square test; ^(3)^ Fisher’s exact test. ACEI: angiotensin-converting enzyme inhibitors; BSA: body surface area; PCI: percutaneous coronary intervention.*

**Table 2:** – Distribution of the ST-segment–elevation myocardial infarction arterial territory

Variables	Control group	Therapy group	p value
ADA	26 (52%)	26 (52%)	0.83 ^(1)^
RCA	14 (28%)	17 (34%)	
CXA	10 (20%)	7 (14%)	

*Variables expressed as number (%). ^(1)^ Chi-square test. ADA; anterior descending coronary artery; CXA: circumflex artery; RCA: right coronary artery.*

Table 3 shows the values of ventricular volumes and LVEF in the total population and in the control and therapy groups, at 72 hours and 6 months after randomization. The group that received sonothrombolysis (therapy group) had lower end-diastolic and end-systolic volumes and higher LVEF than the control group 72 hours after STEMI. All patients underwent RTMPE at follow-up, and this difference was maintained at 6 months of follow-up.

**Table 3:** – Volumes and ejection fraction obtained by real-time myocardial perfusion echocardiography at 72 hours and 6 months after randomization

Variables	Total	Groups		p (between control and therapy groups)

		Control	Therapy	
72 hours				
EDV (mL)	108 ± 35	114 ± 40	102 ± 29	0.096 ^(1)^
ESV (mL)	59 ± 30	66 ± 34	53±23	0.022 ^(1)^
LVEF (%)	47 ± 11	44 ± 11	50±10	0.006 ^(1)^
WMSI	1.68 ± 0.39	1.75 ± 0.40	1.62 ± 0.39	0.09 ^(1)^
Number of segments with perfusion defects	6.42 ± 3.49	5.92 ± 3.47	6.94 ± 3.39	0.15 ^(1)^
6 months				
EDV (mL)	122 ± 47	136 ± 52*	109 ± 36	0.003 ^(1)^
ESV (mL)	66 ± 39	76 ± 45*	55 ± 29	0.006 ^(1)^
LVEF (%)	50 ± 12	47 ± 12*	53 ± 10*	0.008 ^(1)^
WMSI	1.52 ± 0.37	1.64 ± 0.44*	1.46 ± 0.36*	0.02 ^(1)^
Number of segments with perfusion defects	5.86 ± 3.84	6.57 ± 4.29	4.64 ± 3.31*	0.01 ^(1)^

Variables expressed as mean ± standard deviation. ^(1)^ Unpaired Student’s t test between control and therapy groups. *p < 0.05 on paired Student’s t test (comparison of parameters between 6 months and 72 hours). EDV: end-diastolic volume; ESV: end-systolic volume; LVEF: left ventricular ejection fraction; WMSI: wall motion score index.

There were no significant differences between the therapy and control groups in relation to WMSI at 72 hours (1.62 ± 0.39 versus 1.75 ± 0.40; p = 0.09), but, after 6 months of follow-up, the therapy group evolved with lower WMSI than the control group (1.46 ± 0.36 versus 1.64 ± 0.44; p = 0.02), as shown in Figure 4 . The decreased WMSI values demonstrate improved left ventricular function. In relation to myocardial perfusion obtained by RTMPE, no differences were observed between the number of segments with perfusion defects between the therapy and control groups 72 hours after STEMI (5.92 ± 3.47 versus 6.94 ± 3.39; p = 0.15), but, at 6 months of follow-up, the therapy group showed a lower number of segments with perfusion defects than the control group (4.64 ± 3.31 versus 6.57 ± 4.29; p = 0.01), as shown in Figure 5 . In the mean period of 17 months, 8 patients (16%) died in the control group, and 8 patients (16%) died in the therapy group.

**Figure 4:** – Wall motion score index in the control and therapy groups evaluated by real-time myocardial perfusion echocardiography 72 hours and 6 months after randomization.

* Unpaired Student’s t test

**Figure 5:** – Number of segments with perfusion defects in the control and therapy groups evaluated by real-time myocardial perfusion echocardiography 72 hours and 6 months after randomization.

## Discussion

This is the first study in human beings to evaluate the effects of sonothrombolysis on left ventricular function and perfusion at 6 months of follow-up after STEMI. Using RTMPE, we have demonstrated that patients with STEMI treated with this novel therapy showed improvements over time with respect to WMSI and the number of segments with myocardial perfusion defects. The results of the MRUSMI trial demonstrated that door-to-balloon times were not different between the control and therapy groups (78 ± 32 minutes versus 77 ± 26 minutes, respectively; p = 0.42). Recanalization of the culprit vessel at first angiography before primary angioplasty was observed in 24/50 (48%) patients of the therapy group in comparison with 10/50 (20%) in the control group (p < 0.001). On magnetic resonance conducted 72 hours after STEMI, the therapy group showed lower infarct size than the control group (29 ± 22 grams versus 40 ± 20 grams; p = 0.026). ^13^

These beneficial effects were evident on the microvascular level, with improved capillary flow observed immediately after percutaneous coronary intervention. Prior to this, the behavior of the WMSI and the number of segments with myocardial perfusion defects over time had not been evaluated. Our results confirm that the early recanalization and improved coronary microcirculation obtained with sonothrombolysis have additional benefits for patients with STEMI, when compared to patients who received conventional treatment with primary angioplasty.

High-energy transthoracic ultrasound has been studied as an adjunctive treatment to fibrinolytic drugs in the approach to arterial thrombi and as an isolated method for treating vascular thrombi. ^20 , 21^ A proposed mechanism for explaining how ultrasound dissolves thrombi is by inducing cavitation. ^22 , 23^ Cavitation is the ultrasonic generation of gas bodies that expand and retract. This leads to shear forces that disrupt the environment, with the potential to rupture thrombi. Studies using catheter-based systems capable of releasing ultrasound into the coronary artery have proven that they were able to dissolve thrombi without the use of a fibrinolytic agent. This type of high-energy, low-frequency ultrasonic system, with 45 kHz, delivered through the tip of a 1.6-mm catheter has been shown to successfully recanalize the anterior descending artery of patients who had AMI of the anterior wall. ^24^ In order to overcome the limitations of ultrasound in acute coronary syndromes, experimental studies have demonstrated that the association of administration of microbubbles under the effect of ultrasound can accelerate thrombi dissolution. Gas microbubbles are small microspheres that have specific acoustic properties that make them highly useful as ultrasound contrast agents for diagnostic imaging. Because they act as cavitation nuclei, microbubbles reduce the peak negative pressure threshold required to induce cavitation. In this manner, the destruction of microbubbles mediated by ultrasound may further accelerate thrombi dissolution. In animal models of thrombosed iliofemoral arteries, low-frequency transcutaneous ultrasound associated with intravenously injected microbubbles produced recanalization rates above 90%, without requiring a thrombolytic agent. ^25^ In a preclinical study of 45 pigs, it was demonstrated that, during a continuous intravenous infusion of microbubbles containing perfluorocarbon gas, the ultrasonic energy emitted by a diagnostic ultrasound transducer was able to restore microcirculation flow and improve coronary artery recanalization rates. ^26^ The randomized clinical PLUS trial, which attempted to evaluate the additional value of therapeutic ultrasound alone, without microbubbles, in patients with AMI, was interrupted. ^10^ Interruption of the study was recommended in July 2003 due to the low likelihood of significant differences in coronary flow grade on the Trombolysis in Myocardial Infarction score or ST-segment resolution with treatment by ultrasound. We now know that a possible cause of the study’s failure was the fact that it did not associate intermittent ultrasound with microbubbles. Without the latter, there is not enough tissue inertial cavitation and release of nitric oxide to promote sonothrombolysis and reduce no-reflow effectively. ^10^ Mathias et al., ^12^ in 2016, published a pilot study evaluating 30 patients, demonstrating the safety and feasibility of the application of high-MI ultrasound and continuous infusion of microbubbles for early recanalization and improved coronary microcirculation in patients with STEMI. ^12^ These findings were confirmed in the MRUSMI trial, increasing the population to 100 patients. ^13^ The high-MI impulses used to improve epicardial and microvascular recanalization in this study are part of a standard resource in an ultrasound system that is normally used to evaluate myocardial perfusion and regional wall motion. ^14 - 17^ These high-MI impulses cause cavitation in microbubbles (increase and collapse) during the period of insonation that finally ruptures them. ^9^ This growth and collapse cause shear tension in regions close to the microbubbles, which, in the case of a thrombus, results in its dissolution.

The reasons as to why sonothrombolysis can result in improved WMSI and myocardial perfusion in 6 months can be associated with several factors that are not yet fully known. The main factor seems to be the early recanalization of the coronary arteries, before performing percutaneous coronary intervention, as observed in the MRUSMI trial (48% in the therapy group versus 20% in the control group). Moreover, a smaller infarcted area was observed on magnetic resonance at 72 hours in the therapy group. Another possible effect could be related to the induction of nitric oxide release. ^27^ Future multicenter studies are needed to clarify the pathophysiological mechanisms and to prove the benefits of sonothrombolysis in patients with acute coronary syndromes. It is worth underscoring the potential of this new therapeutic option for treating acute thrombotic conditions. ^28 , 29^

### Study limitations

Given that this study was a sub-analysis of the MRUSMI trial, the remaining data pertaining to results related to angiography, electrocardiography, and cardiac biomarkers have been previously reported. Our results were limited to the findings of RTMPE, with a focus on analysis of WMSI and the number of segments with myocardial perfusion defects. That notwithstanding, we emphasize the novelty of these findings, as well as the importance of these effects at 6 months of follow-up in patients with STEMI who were treated by sonothrombolysis. This is a single-center study, with a small number of patients, and it should be extended to multicenter evaluations in order to confirm the findings of this pioneering study. Another point that could be raised as a study limitation is regarding the fact that analysis of wall motion and myocardial perfusion may be considered to be subjective; however, we highlight the widespread application of these scores in routine echocardiography practice, as well as the fact that the researchers involved in this study have ample experience with the technique of RTMPE. ^30 - 32^

## Conclusion

Sonothrombolysis is a new therapeutic approach for treating patients with STEMI that results in improved left ventricular wall motion score and reduced perfusion defects over time.
